# The ecology and evolution of non-domesticated *Saccharomyces* species

**DOI:** 10.1002/yea.3040

**Published:** 2014-10-23

**Authors:** Primrose J Boynton, Duncan Greig

**Affiliations:** 1 Max Planck Institute for Evolutionary BiologyPlön, Germany; 2 Galton Laboratory, Department of Genetics, Evolution and Environment, University College LondonUK

**Keywords:** *Saccharomyces paradoxus*, *S. eubayanus*, *S. uvarum*, *S. kudriavzevii*, *S. arboricola*, *S. mikatae*

## Abstract

Yeast researchers need model systems for ecology and evolution, but the model yeast *Saccharomyces cerevisiae* is not ideal because its evolution has been affected by domestication. Instead, ecologists and evolutionary biologists are focusing on close relatives of *S. cerevisiae*, the seven species in the genus *Saccharomyces*. The best-studied *Saccharomyces* yeast, after *S. cerevisiae*, is *S. paradoxus*, an oak tree resident throughout the northern hemisphere. In addition, several more members of the genus *Saccharomyces* have recently been discovered. Some *Saccharomyces* species are only found in nature, while others include both wild and domesticated strains. Comparisons between domesticated and wild yeasts have pinpointed hybridization, introgression and high phenotypic diversity as signatures of domestication. But studies of wild *Saccharomyces* natural history, biogeography and ecology are only beginning. Much remains to be understood about wild yeasts' ecological interactions and life cycles in nature. We encourage researchers to continue to investigate *Saccharomyces* yeasts in nature, both to place *S. cerevisiae* biology into its ecological context and to develop the genus *Saccharomyces* as a model clade for ecology and evolution. © 2014 The Authors. Yeast published by John Wiley & Sons Ltd.

## Introduction

**Figure 1:**
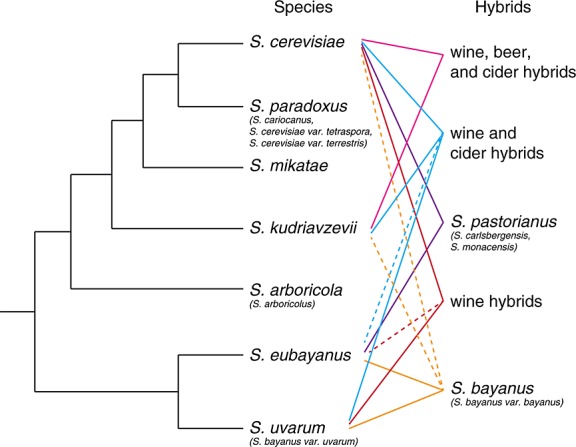
Schematic cladogram depicting phylogenetic relationships among *Saccharomyces* species and well-known or frequently isolated hybrids. Dashed lines represent introgressions from a third or fourth species into a hybrid. Most introgressions are not present in all hybrid strains. Synonyms are given in parentheses below species names. Cladogram topography from Almeida *et al*. (2014; Figure1a)

*Saccharomyces cerevisiae* is arguably the most intensely studied eukaryotic organism besides human beings. Its genetic tractability has made it a valuable model organism for genetics, genomics, cell biology and biochemistry (e.g. Goffeau *et al*., 1996; Spellman *et al.*, 1998; Hartwell *et al.*, 1974). But its long history of human domestication makes it less than ideal for ecology and evolution research. Evolutionary biologists and ecologists often prefer to study other species in the genus *Saccharomyces*, which comprises seven known species and many hybrids (Figure1). All are as tractable as *S. cerevisiae* in the laboratory. Several, including *S. cerevisiae*’s closest relative, *S. paradoxus*, are found only in the wild and not in human fermentations. All *Saccharomyces* species have similar morphologies and biochemical phenotypes (Vaughan-Martini and Martini, 2011), although there are some ecologically significant traits that differ among species, e.g. temperature tolerance (Sampaio and Gonçalves, 2008). Information about *Saccharomyces* yeasts can put *S. cerevisiae* molecular biology into ecological and evolutionary context. This clade has also taught us lessons about niche ecology, hybridization, domestication, population genetics and biogeography that go beyond comparisons with *S. cerevisiae*.

Here we will review the use of *S. paradoxus* and its relatives to understand yeast natural history, ecology and evolution. We focus on *S. paradoxus* because it is the best-studied *Saccharomyces* yeast besides *S. cerevisiae*. The literature on *Saccharomyces* species that are not *S. paradoxus* or *S. cerevisiae* is growing quickly, and we include information on other *Saccharomyces* species (*S. eubayanus*, *S. uvarum*, *S. kudriavzevii*, *S. arboricola* and *S. mikatae*) when it is available. We recommend additional recent *Saccharomyces* reviews that focus on comparisons with *S. cerevisiae* (Replansky *et al.*, 2008), speciation (Greig, 2009) and evolutionary genomics (Hittinger, 2013) for interested readers.

## History and taxonomy

The needs of brewing and winemaking motivated the study of *Saccharomyces* yeasts in the nineteenth and twentieth centuries. The genus name *Saccharomyces* was first used to describe fermentation yeasts in the early nineteenth century (Meyen, 1839). As the industrial revolution progressed, attempts were made to improve beer production, consistency and shelf-life. The French scientist Louis Pasteur (1879) developed methods to keep beer free of contaminating moulds and bacteria, and he distinguished strains used for making traditional top-fermented ales from those used to make German bottom-fermented lagers (now named *S. cerevisiae* and *S. pastorianus*, respectively). In revenge for the Franco-Prussian war, Pasteur did not permit his methods to be translated into German, instead using them to promote the competing French brewing industry (Baxter, 2001). Pasteur's work influenced the development of a new Danish industrial brewer, Carlsberg. Emil Christian Hansen (1896), working in the Carlsberg Laboratories, developed single-colony culturing methods, and his successor, Øjvind Winge, pioneered the science of yeast genetics in the early twentieth century (Szybalski, 2001).

Over the course of the twentieth century, the genus *Saccharomyces* was revised several times. Researchers added and removed many taxa that are now placed in other genera related to *Saccharomyces* (‘*Saccharomyces sensu lato*’, in contrast with ‘*Saccharomyces sensu stricto*’, which are taxa currently assigned to the genus *Saccharomyces*) (Kurtzman, 2003). Taxonomists also described new *Saccharomyces* species based on carbon and nitrogen assimilation tests. Many newly described *Saccharomyces* species later turned out to be phenotypically divergent strains of previously described species (Vaughan-Martini and Martini, 1995; Naumov, 1996). Throughout the twentieth century, almost all known *Saccharomyces* species came from human-associated fermentations.

**Figure 2:**
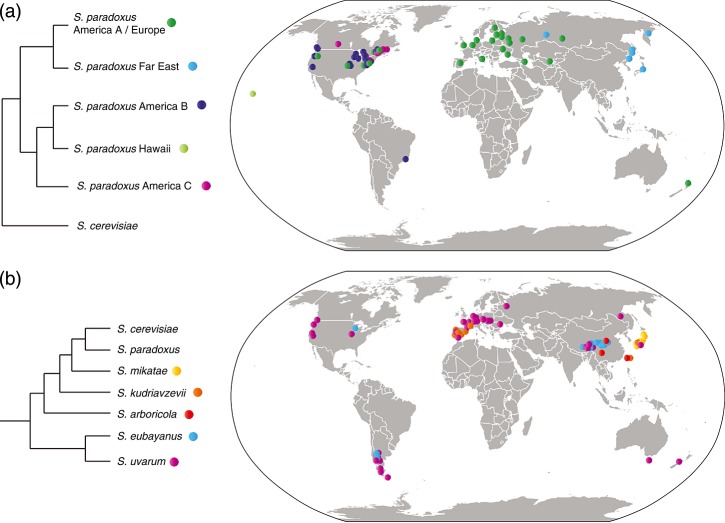
Locations from which *Saccharomyces* yeasts have been isolated: (a) *S. paradoxus* populations; (b) *Saccharomyces* species, not including *S. paradoxus* and *S. cerevisiae*. Dots represent isolation locations and colours represent species or populations; locations are approximate; overlapping dots of different colours are offset for clarity; where the only available location data available is the name of a country, dots are placed near the country's centre (location data from National Institute of Technology and Evaluation, 2014; Zhang *et al.*, 2010; Erny *et al.*, 2012; Kuehne *et al.*, 2007; Naumov *et al.*, 1997, 2000, 2013; Liti *et al.*, 2009; LeDucq *et al.*, 2014; Wang and Bai, 2008; Sampaio and Gonçalves, 2008; Lopes *et al.*, 2010; Almeida *et al.*, 2014; Bing *et al.*, 2014; Hyma and Fay, 2013; and papers cited therein). *S*. *paradoxus* cladogram redrawn from Liti *et al*. (2009) and LeDucq *et al.* (2014). Map graphic altered from https://en.wikipedia.org/wiki/File:BlankMap-World8.svg, accessed 13 June 2014

*S. paradoxus* was the first *Saccharomyces* yeast to be acknowledged as a non-domesticated species. It was first isolated from sap exudate of a tree in Russia (Batshinskaya, 1914). Subsequent isolates described as *S. cerevisiae* var. *tetraspora*, *S. cerevisiae* var. *terrestris*, *S. cariocanus* and other synonyms have been reidentified as *S. paradoxus*, based on genomic sequence data, DNA–DNA hybridization or by mating with *S. paradoxus* tester strains (Liti *et al.*, 2006; Vaughan-Martini, 1989; Naumov, 1996). Starting in the 1980s and continuing to the present, researchers isolated and re-identified many *S. paradoxus* strains from tree bark, soil and other substrates throughout the world (Figure2a) (Naumov *et al.*, 1998; Vaughan-Martini, 1989; Sniegowski *et al.*, 2002). The high frequency of *S. paradoxus* isolation in nature inspired many researchers to look for other naturally occurring *Saccharomyces* species and to use modern genetic analyses to identify them.

Early in the twenty-first century, researchers described the naturally occurring species *S. kudriavzevii*, *S. mikatae*, *S. arboricola* and *S. eubayanus* in quick succession. *S. kudriavzevii* and *S. mikatae* strains from decayed leaves and soil were described from a Japanese culture collection (Naumov *et al.*, 2000). A few years later, *S. arboricola* (syn: *S. arboricolus*) was discovered on hardwood bark in China (Wang and Bai, 2008). Researchers have since identified a few more *S. mikatae* and *S. arboricola* strains from Japan and Taiwan, although these two yeasts have never been isolated outside of eastern Asia (Naumov *et al.*, 2013; National Institute of Technology and Evaluation, 2014). In contrast, shortly after the discovery of *S. kudriavzevii* in Japan, researchers identified a European population of *S. kudriavzevii* (Sampaio and Gonçalves, 2008). The European population includes newly discovered *S. kudriavzevii*/*S. cerevisiae* and *S. kudriavzevii*/*S. cerevisiae*/*S. uvarum* hybrids from wine, beer and cider fermentations (Figure1) (Lopandic *et al.*, 2007; González *et al*., 2008; Sipiczki, 2008).

*S. eubayanus*, the most recently discovered *Saccharomyces* species, is one parent of the lager-brewing hybrid species *S. pastorianus* (Figure1) (Libkind *et al.*, 2011). For decades, taxonomists recognized *S. pastorianus* (syn: *S. carlsbergensis*, *S. monacensis*) as a hybrid of *S. cerevisiae* and another yeast, but had difficulty identifying the second parent (Nguyen and Gaillardin, 2005; Nguyen *et al.*, 2011). Candidates included the species currently named *S. bayanus* and *S. uvarum*, but none sufficiently matched the non-*S. cerevisiae* portion of *S. pastorianus* genomic DNA. The discovery of *S. eubayanus* associated with southern beech trees in South America solved the mystery of *S. pastorianus* parentage. *S. eubayanus* genomic DNA is over 99% similar to the non-*S. cerevisiae* portion of *S. pastorianus* genomic DNA (Libkind *et al.*, 2011). More strains of *S. eubayanus* were subsequently found associated with trees in Tibet, Sichuan and western China; relatives of Tibetan *S. eubayanus* are likely parents of *S. pastorianus* (Bing *et al.*, 2014). Both before and since the discovery of *S. eubayanus*, *S. pastorianus* has been used as a model organism to study hybridization's impact on genomes and phenotypes (reviewed by Gibson and Liti, 2014).

Questions remain about the origin of the lager yeast *S. pastorianus*. Lager beer is fermented and stored at low temperatures, and was first produced in Bavaria in the sixteenth century or earlier (Lager is the German word for a store or warehouse). Lager is now the most popular style of beer in the world (84% of the global beer market; Marketline, 2013). While records of brewing practices in Europe between the sixteenth and nineteenth centuries exist (reviewed in Meussdoerfer, 2009), there are no genetic or taxonomic data identifying lager yeast species before the nineteenth century, when *S. pastorianus* was identified (Pasteur, 1879; Hansen, 1896). It is not known whether *S. pastorianus* was used to produce the earliest lagers, or whether *S. bayanus*, *S. uvarum*, cold-tolerant *S. cerevisiae* strains or another yeast was originally used. The timing and circumstances of the *S. pastorianus* hybridization event are also unknown. Libkind *et al.* (2011) hypothesized that early trans-Atlantic traders introduced *S. eubayanus* into the European brewing environment from South America, where it hybridized with *S. cerevisiae*. Alternatively, Bing *et al.* (2014) hypothesized that *S. eubayanus* was introduced to Europe from Tibet via the Silk Road. The Silk Road hypothesis is favoured because non-*S. cerevisiae S. pastorianus* genes have higher sequence similarity with Tibetan *S. eubayanus* than with South American *S. eubayanus*, based on multilocus sequencing [99.8% sequence similarity compared to 99.4% (Bing *et al.*, 2014); note that a whole-genome sequence-based estimate of similarity between South American *S. eubayanus* and the non-*S. cerevisiae* portion of *S. pastorianus* is slightly higher at 99.6% (Libkind *et al.*, 2011): whole-genome sequences are not yet available for Asian *S. eubayanus*]. We propose two additional hypotheses concerning the *S. pastorianus* hybridization event: *S. eubayanus* may have existed in wild European populations when lager brewing was developed, or *S. eubayanus* may have been introduced from China or Tibet relatively recently, while brewers used a non-*S. pastorianus* yeast to produce lager beer. Further archaeological and historical study, paired with DNA analysis, is needed to definitively identify the yeast responsible for brewing the first lagers and the circumstances surrounding the *S. pastorianus* hybridization event.

The discovery of *S. eubayanus* also enabled taxonomists to characterize *S. uvarum* and another hybrid species, *S. bayanus*. Both are associated with human fermentations, including beer, cider and wine (Pérez-Través *et al*., 2014; Nguyen *et al.*, 2011; Almeida *et al.*, 2014). *S. uvarum* also occurs on hardwood bark, soil and insects, and often co-occurs with *S. eubayanus* (Almeida *et al.*, 2014). Genomic comparisons revealed *S. uvarum* to be the sister species of *S. eubayanus*, and *S. bayanus* to be a hybrid between *S. uvarum* and *S. eubayanus* (Peris *et al.*, 2014; Pérez-Través *et al.* 2014). Prior to the discovery of *S. eubayanus*, taxonomists considered both *S. uvarum* and *S. bayanus* to be varieties of the species *S. bayanus* (*S. bayanus* var. *uvarum* and *S. bayanus* var. *bayanus*, respectively; Vaughan-Martini and Martini, 2011) because they were phenotypically and genetically similar. Many strain collections and databases have not yet been updated, and we caution researchers to verify strain identities when using publicly available data. For example, the first sequenced *S. uvarum* genome is currently identified in the NCBI database as *S. bayanus* strain MCYC 623 (Cliften *et al.*, 2003; NCBI assembly Accession No. ASM16699v1, accessed 8 July 2014).

Four of the seven known *Saccharomyces* species were discovered in the last 20 years, and we expect researchers to continue to discover new species in the near future. Apart from *S. paradoxus*, all the known *Saccharomyces* species that are not associated with human fermentations are recent discoveries. Eastern Asia may be a centre of diversity of the genus. *S. arboricola*, *S. mikatae* and *S kudriavzevii* were all discovered in Japan or China, and China is a centre of genetic diversity for *S. cerevisiae* and *S. eubayanus* (Wang *et al.*, 2012; Bing *et al.*, 2014). In addition, researchers are beginning to investigate undersampled locations: *S. eubayanus* was discovered in Argentina, and subsequent sampling throughout Argentina uncovered diverse *S. uvarum* populations (Libkind *et al.*, 2011; Almeida *et al.*, 2014).

## Ecology and natural history

*Saccharomyces* yeasts are most often found associated with hardwood bark, soil and leaf surfaces (e.g. Sniegowski *et al.*, 2002; Glushakova *et al.*, 2007; Libkind *et al.*, 2011; Wang *et al.*, 2012; Wang and Bai, 2008). The most frequent *Saccharomyces* hosts are oak trees (*Quercus* spp.) in the northern hemisphere, and southern beech trees (*Nothofagus* spp.) in the southern hemisphere (Sampaio and Gonçalves, 2008; Naumov *et al.*, 1998; Almeida *et al.*, 2014; Peris *et al.*, 2014). *S. eubayanus* and *S. uvarum* were also recently isolated from *Araucaria araucana*, a South American gymnosperm (Rodríguez *et al*., 2014). *Saccharomyces* dispersal between substrates is poorly understood. *S. paradoxus* and *S. uvarum* have occasionally been isolated from insects, including *Drosophila* spp. (Naumov *et al.*, 2000; Ivannikova *et al.*, 2006). Stefanini *et al.* (2012) proposed insects as a *S. cerevisiae* dispersal vector in vineyards, and insects may disperse other *Saccharomyces* species.

Sampling biases may give an incomplete picture of *Saccharomyces* habitats and ranges. Researchers use enrichment culture to isolate *Saccharomyces* spp. from nature (e.g. Sampaio and Gonçalves, 2008; Naumov *et al.*, 1998): a sample of bark or soil is incubated in high-sugar liquid medium, with or without added acid, ethanol or antibiotics to control bacterial growth. After a few days to a few weeks, a portion of the enrichment medium is streaked onto solid medium. Individual colonies are identified morphologically and using DNA sequencing. It is not known whether samples that fail to yield *Saccharomyces* do not contain *Saccharomyces*, or whether other microbes outcompete existing *Saccharomyces* cells in a sample. Such false-negative enrichment cultures could give an inaccurate impression of the distribution or abundance of a species. For example, the apparent association of *S. paradoxus* with oak trees could be a result of absence of microbes on oak bark that grow well in enrichment medium. The problem is well illustrated by the observation that the same sampling scheme tends to recover *S. paradoxus* and *S. cerevisiae* when enrichment cultures are incubated at 30 °C, and *S. uvarum* and *S. kudriavzevii* at 10 °C (Sampaio and Gonçalves, 2008). Another factor is that sampling sites may not be chosen systematically. Until recently, sampling effort has been concentrated in the northern hemisphere, especially Europe, North America and Japan (Figure2). In the past 4 years, intensive sampling uncovered *S. uvarum* and *S. eubayanus* populations in Argentina and China (Almeida *et al.*, 2014; Bing *et al.*, 2014; Peris *et al.*, 2014). Insufficient sampling may be the reason why there are no known *Saccharomyces* isolates from Africa (besides human-associated *S. cerevisiae*; e.g. Liti *et al.*, 2009; Legras *et al.*, 2007; Naumov and Naumova, 2011); we consider African hardwoods to be a likely *Saccharomyces* habitat, and future sampling in Africa may reveal undiscovered *Saccharomyces* populations or species.

The apparent association of wild *Saccharomyces* yeasts with bark, soil and leaves is unexpected, because *Saccharomyces* yeasts grow on high-sugar substrates when they associate with humans. *Saccharomyces* yeasts are Crabtree-positive, i.e. they ferment when glucose concentrations are high, even when oxygen is available for more efficient aerobic respiration. The Crabtree effect is a hypothesized adaptation to competition on high-sugar substrates such as fruit, because Crabtree-positive yeasts can exploit sugars more quickly than Crabtree-negative competitors (Piškur *et al*., 2006). Paradoxically, *Saccharomyces* yeasts are rarely found on fruit in nature and instead most frequently associate with bark. There are several possible explanations for the presence of *Saccharomyces* on bark. *Saccharomyces* yeasts may be contaminants from a nearby sugar-rich substrate; e.g. *S. eubayanus* and *S. uvarum* have been isolated from *Cyttaria* galls on *Nothofagus* and from *Nothofagus* bark (Libkind *et al.*, 2011; Almeida *et al.*, 2014). *Cyttaria* is a biotrophic *Nothofagus* parasite, and *Cyttaria*-infected trees produce sugar-rich galls (Libkind *et al.*, 2011). In addition, many researchers have specifically targeted sugar-rich oak exudates when sampling for *Saccharomyces* (Naumov *et al.*, 1998). In both cases, it is possible that yeasts on bark or soil are contaminants from high-sugar gall or exudate environments. Another possible explanation is that yeasts normally grow on trace amounts of hexose sugars or other nutrients present on bark surfaces (Sampaio and Gonçalves, 2008), and competitive mechanisms other than the Crabtree effect are responsible for their success. Alternatively, both environments may form part of the *Saccharomyces* natural habitat: bark may provide a natural refuge when fruit is not available; or wild *Saccharomyces* might be ubiquitous generalists, able to grow and survive in a wide range of habitats and conditions. Researchers must further investigate yeast behaviour on oak and soil substrates, in addition to high-sugar laboratory media or fruit juices, to understand yeast ecological realities and selection pressures.

Multiple *Saccharomyces* species can co-occur in a habitat, and temperature niche partitioning is the best-studied explanation for co-occurrence. Reproductively isolated *Saccharomyces* can appear on the same tree, sometimes within centimetres of one another (Kuehne *et al.*, 2007). Pairs of species that frequently co-occur include *S. cerevisiae*/*S. paradoxus*, *S. eubayanus*/*S. uvarum*, *S. kudriavzevii*/*S. paradoxus* and *S. uvarum*/*S. paradoxus* (Sniegowski *et al.*, 2002; Sampaio and Gonçalves, 2008; Libkind *et al.*, 2011; Bing *et al.*, 2014; Hyma and Fay, 2013). Diverged and reproductively isolated *S. paradoxus* populations can also co-occur (Kuehne *et al.*, 2007; LeDucq *et al.*, 2014). Co-occurring species or populations often have clearly different growth temperature optima. For example, pairs or triplets of yeast species from Portuguese oak trees, e.g. *S. paradoxus* and *S. kudriavzevii*, have maximum growth temperatures different from one another. Thermo- and cryotolerance correlate with glycolysis protein sequence, and glycolysis may be a key pathway in temperature adaptation (Gonçalves *et al*., 2011). Co-occurring *Saccharomyces* species may temporally partition temperature niches between day time and night time, or among seasons of the year, with one active species during cold times and another during warm times (Gonçalves *et al.*, 2011).

*Saccharomyces* species may also partition niches besides, or in addition to, temperature. For example, while different North American *S. paradoxus* populations have different ranges and temperature tolerances, with coexistence at range edges, temperature tolerance does not exactly correlate with population range temperatures (Figure2) (LeDucq *et al.*, 2014). In addition, the cryotolerant yeasts *S. uvarum* and *S. eubayanus* have been isolated from the same locations in Argentina and China (Libkind *et al.*, 2011; Bing *et al.*, 2014). In both cases, yeasts may partition more than one niche, or neutral or dispersal effects may be responsible for local *Saccharomyces* diversity.

## Life cycles

In the laboratory (and presumably in nature), *Saccharomyces* life cycles resemble those of *S. cerevisiae* (Vaughan-Martini and Martini, 2011; Tsai *et al.*, 2008; *S. cerevisiae* life cycle reviewed in Herskowitz, 1988). Yeasts engage in sexual and asexual reproduction, and sexual reproduction includes inbreeding and outbreeding. Briefly, diploid cells reproduce mitotically in nutrient-rich media but, when starved, diploid cells undergo meiosis to produce one to four haploid spores (ascospores) enclosed within a sac (ascus, plural asci; most asci contain a meiotic tetrad of four ascospores, two of each mating type). Ascospores are resistant to environmental stresses, including conditions within insect digestive tracts (Coluccio *et al.*, 2008; Reuter *et al.*, 2007). When nutrients are restored, ascospores can germinate into haploid cells. A haploid cell can reproduce mitotically, but will usually fuse with another haploid cell of the opposite mating type to form a diploid vegetative cell soon after germination. Most mating occurs between haploids produced from the same meiosis, a form of self-fertilization known as intra-tetrad mating, or automixis. Mating can also occur between haploids from different tetrads, which can be more or less related (inter-tetrad mating). Haploids that have already undergone mitosis can switch mating type at the following mitotic division, allowing them to mate with their clone-mates (autodiploidization) to form perfectly homozygous diploids.

There is contradictory information on the relative amounts of inbreeding and outbreeding in *S. paradoxus*. Population genetic and genomic data suggest that European and Far Eastern *S. paradoxus* go through a sexual cycle once every 1000 asexual generations, and that for each sexual cycle, 94% of matings are intra-tetrad, 5% are autodiploidization and 1% are inter-tetrad (Tsai *et al.*, 2008). This estimate of one inter-tetrad mating/10^2^ sexual cycles or 10^5^ mitotic divisions is based on comparisons of mutation-based and recombination-based effective population size estimates, as well as calculations of linkage disequilibrium at different distances from the mating type locus along the chromosome. A similar estimate of one outcrossing event/5 × 10^4^ *S. cerevisiae* mitoses was calculated by inferring recombination events from discordant phylogenies (Ruderfer *et al.*, 2006). In contrast to these population genetic estimates, laboratory observations of wild *S. paradoxus* strains show that inter-tetrad mating rates can be surprisingly high (11–43% of matings; Murphy and Zeyl, 2010). We expect inter-tetrad mating to produce outcrossed progeny because different *S. paradoxus* genotypes live in close proximity to one another. For example, different *S. paradoxus* genotypes exist within 5 cm of one another on oak trees in the UK, and *S. paradoxus* from different populations exist within the same 100 cm^2^ sampling area on North American trees (Koufopanou *et al.*, 2006; Kuehne *et al.*, 2007). Based on laboratory observations, we suspect that natural outbreeding rates may be higher than population genetic estimates suggest. However, laboratory observations are limited because yeast behaviour may be different in natural conditions. Population genetic estimates are also limited because they rely on assumptions that are difficult to evaluate, e.g. that mutation rates are the same in the laboratory and in nature; that mutation and recombination rates are the same in *S. cerevisiae* and *S. paradoxus*. Resolving the contradiction between the very low outbreeding rates estimated by population genetics methods and high inter-tetrad mating rates observed in the laboratory will require more research.

Hybridization and introgression events occasionally occur among *Saccharomyces* species, and domestication appears to select for hybrid genomes. Haploid cells from different *Saccharomyces* species can mate in the laboratory to form F1 hybrids, which grow normally by mitosis. However, when meiosis is induced, chromosomes from different species fail to recombine and cannot segregate efficiently; 99% or more of the resulting ascospores lack essential chromosomes and are non-viable (Hunter *et al.*, 1996; Greig *et al.*, 2002a). Different *Saccharomyces* are thus post-zygotically reproductively isolated. The few viable spores that survive an F1 hybrid meiosis contain a variable and usually aneuploid mixture of chromosomes from both parental species, but can mate to form F2 hybrids (Greig *et al.*, 2002b). Many spontaneously occurring two- and three-way hybrids have been found in wine, cider and beer (Figure1) (Lopandic *et al.*, 2007; González *et al.*, 2008; Sipiczki, 2008). The most famous fermentation hybrids are the two-way hybrids *S*. *pastorianus* and *S. bayanus* (Pérez-Través *et al.*, 2014; Nguyen *et al.*, 2011). *Saccharomyces* hybrids are rare outside of fermentation environments, but a few putative hybrids have been reported between *S. paradoxus* and *S. cerevisiae*, and one has been reported between *S. paradoxus* and *S. kudriavzevii* (Zhang *et al.*, 2010; Liti *et al.*, 2005). Further research is needed to confirm the extent of hybridization or introgression in these naturally occurring strains. Laboratory-produced hybrids tend to have higher fitness than their parents in extremely stressful environments, suggesting that hybrids are more common in domesticated than wild environments because domestication imposes novel stresses (Stelkens *et al.*, 2014).

Portions of a chromosome can also introgress from the genome of one *Saccharomyces* species to another. Introgression is most likely the result of a hybridization event followed by many backcrosses to one parent (Liti *et al.*, 2006). Introgressions into fermentation strains are common, and have been documented from *S. paradoxus* into *S. cerevisiae*, *S. cerevisiae* and *S. kudriavzevii* into *S. bayanus*, several species (*S. cerevisiae*, *S. eubayanus* and *S. kudriavzevii*) into *S. uvarum*, and *S. eubayanus* into *S. cerevisiae*/*S. uvarum* and *S. cerevisiae*/*S. kudriavzevii*/*S. uvarum* hybrids (Figure1; Pérez-Través *et al.*, 2014; Doniger *et al.*, 2008; Muller and McCusker, 2009; Almeida *et al.*, 2014; Naumova *et al.*, 2011). Introgressions into fermentation strains are usually present in a subset of strains in a species, and are not fixed in the entire species. Introgressions in naturally occurring strains have rarely been documented. A 23 kb long (12 open reading frames) portion of chromosome XIV from *S. cerevisiae* has introgressed into one *S. paradoxus* population (America A/Europe, see discussion on *S. paradoxus* population structure below). The introgression appears to be fixed in the America A/Europe *S. paradoxus* population but not present in other *S. paradoxus* populations (Liti *et al.*, 2006). In addition, genomes of a few *S. uvarum* strains from natural habitats contain introgressions from *S. cerevisiae*, *S. eubayanus* and/or *S. kudriavzevii* (Almeida *et al.*, 2014). All *S. uvarum* strains with introgressions are human-associated or close relatives of human-associated strains; introgressed strains isolated from natural habitats may have escaped fermentation habitats. A pattern of frequent hybridization and frequent, unfixed introgression events in domesticated environments suggests that selection in domestication environments is extreme and variable.

## Biogeography of non-domesticated *Saccharomyces*

Dispersal limitation and geographic distance impose structure on *S. paradoxus* populations. *S. paradoxus* ranges throughout the northern hemisphere, with additional isolates from South America and New Zealand (Figure2a). DNA sequence divergence of up to about 4% partitions known *S. paradoxus* isolates into five populations that began to diversify between 0.1 and 1 million years ago: Far East, Hawaii, America A/Europe, America B and America C (Table1; Liti *et al.*, 2006; Liti *et al.*, 2009; LeDucq *et al.*, 2014; note that the Hawaiian population is represented by a single strain, and may be a mosaic strain or other outlier). For comparison, *S. paradoxus* and *S. cerevisiae* are diverged by about 14% and are thought to share a common ancestor between 0.4 and 3 million years ago (Liti *et al.*, 2006; 2009). *S*. *paradoxus* populations are generally restricted to single continents, with some exceptions discussed below. Within populations, sequence similarity decays with physical distance on individual oak trees, among trees in a forest and among sites within a continent (Koufopanou *et al.*, 2006). Increasing genetic distance over space is a signature of dispersal limitation.

**Table:** Population divergences within *Saccharomyces* species

Species	Intraspecies genetic or genomic variation (%)	Number of known populations	Information used to calculate variation*	References
*S. paradoxus*	3.8	5	Whole-genome sequences	Liti *et al.*, 2009; LeDucq *et al*., 2014
*S. cerevisiae*	1.4	13	Sequences of nine genes and four intergenic sequences	Wang *et al.*, 2012
*S. kudriavzevii*	4.1†	3	Whole-genome sequences†	Hittinger *et al.*, 2010
*S. arboricola*	Not available	1 or 2	None	Naumov *et al.*, 2013
*S. mikatae*	Not available	1	None	Naumov *et al.*, 2000
*S. eubayanus*	6.02–7.57	5	Sequences of nine genes and three intergenic sequences	Bing *et al.*, 2014; Peris *et al*., 2014
*S. uvarum*	4.4	3	Whole-genome sequences	Almeida *et al.*, 2014

* Readers should use caution when comparing intraspecies genetic variation between studies using multilocus sequencing and those using whole-genome sequencing.

† Nucleotide divergence was calculated for this review from genomic data produced by Hittinger *et al.* (2010). Jukes-Cantor corrected nucleotide divergence was calculated for concatenated contigs. Ambiguous bases and indels were not included.

Diverging *S. paradoxus* populations may be at an early stage of speciation. Haploids from different populations can mate and the resulting diploids grow normally by mitosis. However, up to 86% of the haploid spores produced by these F1 diploids are non-viable for the same reasons that hybrid spores are non-viable: diverged chromosomes fail to segregate properly (Charron *et al.*, 2014; Greig *et al.*, 2003; Kuehne *et al.*, 2007; Liti *et al.*, 2006). But when *S. paradoxus* isolates from South America are crossed with North American isolates from the America B population, up to 95% of the resulting spores are non-viable, even though the two populations are closely related (only 0.3% sequence divergence; Liti *et al.*, 2006, 2009). Four reciprocal translocations in South American isolates are responsible for this reproductive isolation. Researchers using the biological species concept have therefore named South American isolates ‘*S. cariocanus*’, but we and other researchers prefer to include the few South American isolates within *S. paradoxus* (Naumov *et al.*, 2000, 2013; Liti *et al.*, 2006).

Secondary introductions have increased the range of at least one *S. paradoxus* population (America A/Europe) and may have influenced the biogeography of others (Figure2a). Currently, America A/Europe is found across Europe and in at least one site in New Zealand. It is also found in north-eastern North America, sympatric with and reproductively isolated from other American *S. paradoxus* populations (America B and America C; Kuehne *et al.*, 2007; LeDucq *et al.*, 2014). America A/Europe isolates in New Zealand and North America most likely migrated out of Europe recently with respect to the timescale of genetic divergence (Zhang *et al.*, 2010; Kuehne *et al.*, 2007). Low genetic diversity in North American America A/Europe isolates compared to those from Europe further supports the hypothesis that this population was introduced from Europe to North America after it had diversified in Europe. We do not yet know how European *S. paradoxus* arrived in North America, but human beings probably introduced *S. paradoxus* to New Zealand: isolates have been found on introduced oaks, including acorns, but not native southern beech. Humans may have introduced *S. paradoxus* to New Zealand with oak trees from Australia or the UK during the nineteenth century (Zhang *et al.*, 2010).

America B and America C *S. paradoxus* are a final example of potential allopatric divergence and secondary contact. All American *S. paradoxus* populations are currently sympatric in North America and reproductively isolated from one another (Figure2a). America B populations tend to live in warmer habitats than America C populations (LeDucq *et al.*, 2014). Present-day sympatry may be a result of secondary contact after a past event that permitted adaptation to warmer (America B) and cooler (America C) North American climates. Among North American strains, hybrid spore non-viability correlates with both DNA sequence divergence and differences in chromosomal structure. In addition, America B and America C have high within-population variation in chromosome structure, and hybrid spore non-viability correlates with chromosomal changes within these populations (Charron *et al.*, 2014). Population subdivision may be ongoing within America B and America C. Present-day reproductive isolation among North American populations may maintain separate populations in sympatry, which could eventually lead to complete speciation within *S. paradoxus*. North American *S. paradoxus* will give researchers the opportunity to study speciation processes before and during speciation events.

**Figure 3:**
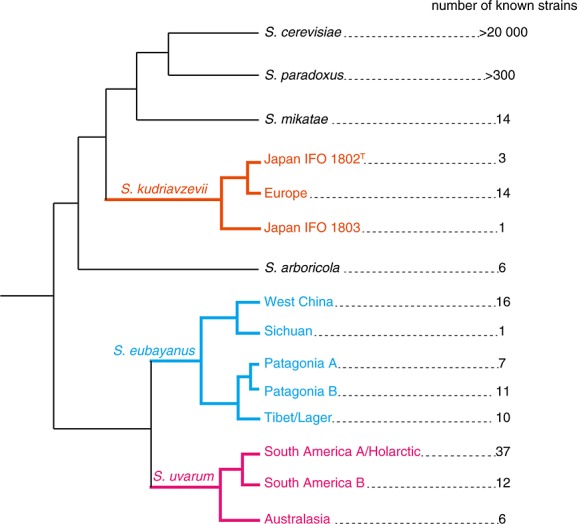
Cladogram depicting *S. kudriavzevii*, *S. eubayanus*, and *S. uvarum* population structure. Strain counts do not include hybrids or strains with mosaic genomes (population data from Hittinger *et al.*, 2010; Bing *et al.*, 2014; Peris *et al.*, 2014; Almeida *et al.*, 2014; F Bai, personal communication: strain count data from population data references and also National Institute of Technology and Evaluation, 2014; Naumov *et al.*, 2013; Wang and Bai, 2008; Liti *et al.*, 2009; LeDucq *et al.*, 2014; Naumov *et al.*, 1997; Kuehne *et al.*, 2007; Zhang *et al.*, 2010; Hyma and Fay, 2013; American Type Culture Collection, 2014)

Like *S. paradoxus*, *S. eubayanus* populations have high genetic diversity and a strong population structure. There are at least five *S. eubayanus* populations: West China, Sichuan, Tibet/Lager, Patagonia A and Patagonia B (Figures2b, and 3) (Bing *et al.*, 2014; Peris *et al.*, 2014). Additional isolates have also been found in Wisconsin, with genomes that are mosaics of Patagonia A and Patagonia B (Peris *et al.*, 2014). The West China and Sichuan populations are diverged and partially reproductively isolated from all other populations (about 7% nucleotide divergence and up to 82% spore non-viability; Table1; Bing *et al.*, 2014). Note that nucleotide divergence within *S. eubayanus* was estimated using multilocus sequencing, which may overestimate divergence; e.g. multilocus sequence-based estimates of divergence between *S. eubayanus* and *S.uvarum* are higher than whole genome-based estimates (9.3–10.3% vs. 6.9%, respectively; Bing *et al.*, 2014; Libkind *et al.*, 2011). Nonetheless, *S. eubayanus* genetic diversity is higher within East Asia than elsewhere. More sampled strains, as well as full genomic data, are needed to understand the implications of *S. eubayanus* population structure for long-distance dispersal and speciation. For example, why are strains from relatively close locations (Tibet and Western China) more highly diverged than Tibetan, Argentinian and Wisconsin strains from a broad geographic area? Is *S. eubayanus* speciating in East Asia, and are speciation mechanisms the same in Asian *S. eubayanus* and North American *S. paradoxus*?

Genetic divergence between *S. kudriavzevii* populations from Europe and Japan (including the type strain, IFO 1802^T^) is about 1%, except for a single Japanese isolate which is much more diverged (IFO 1803, diverged by about 4%; Figure3, Table1). IFO 1803 is likely part of a second Japanese *S. kudriavzevii* population. A remarkable feature of all Japanese strains is that they have completely lost function at seven unlinked *GAL* loci encoding the galactose utilization pathway, a pathway that is functionally maintained in the European population. Analysis of the sequence degradation of the Japanese *gal* pseudogenes indicates that they are nearly as old as the *S. kudriavzevii* lineage itself (Hittinger *et al.*, 2010). The high sequence divergence between Japanese and European *GAL* loci extends into flanking regions, decaying towards the genome-wide average with increasing map distance. This indicates that natural selection maintains the functional and non-functional alleles as separate sets, despite gene flow across the rest of the genome. One explanation is that the sets represent co-adapted gene complexes, and individuals with a mixture of functional and non-functional genes have lower fitness than those with either full sets of non-functional alleles or full sets of functional alleles at all seven loci. The observation that the non-functional set of alleles is present in both unrelated Japanese populations but not in the more closely related European population is also consistent with the possibility that the polymorphism is maintained by local adaptation, rather than co-adapation. Recent sampling has since uncovered French *S. kudriavzevii* strains and European *S. kudriavzevii*/*S. cerevisiae* hybrids that are genetically diverged from the European and Japan IFO 1802^T^ populations, but their *GAL* genotypes have not yet been reported (Erny *et al.*, 2012).

## The effect of domestication on biogeography

Domestication tends to increase phenotypic diversity, e.g. morphological diversity in dogs (Wayne, 1986). *S. cerevisiae* has higher phenotypic diversity but lower genome sequence diversity than *S. paradoxus* (Table1) (Liti *et al.*, 2009; Warringer *et al.*, 2011; Wang *et al.*, 2012). *S*. *cerevisiae* strains also have high variation in gene content, e.g. the presence and absence of genes and/or copy number variations (Bergström *et al.*, 2014). High phenotype diversity may be due to independent domestication of different *S. cerevisiae* founder populations; domestication could relax stabilizing selection, allowing loss or gain of genes and functions by drift that would normally be maintained in the wild (Warringer *et al.*, 2011). Alternatively, different domesticated environments, e.g. rice wine, grape wine or beer, may select directly for different traits. Genetic and phenotypic comparisons were made as part of the *Saccharomyces* Genome Resequencing Project (SGRP): researchers sequenced the genomes of 35 *S. paradoxus* strains from four populations and compared them with 36 *S. cerevisiae* genomes from five populations (Liti *et al.*, 2009; Bergström *et al.*, 2014).

Domestication increases dispersal and reduces geographic structure. In domesticated *S. cerevisiae*, genetic structure is weak and tends to track human usage, and most lineages are mosaics (Liti *et al.*, 2009). In contrast, wild *S. cerevisiae* isolates from primeval Chinese forests show strong geographic structure (Wang *et al.*, 2012). Domestication has affected *S. uvarum* in the same way. *S. uvarum* has only been isolated from natural substrates in the southern hemisphere, while northern hemisphere samples include domesticated and natural isolates. There are three *S. uvarum* populations: Australasia, South America B and South America A/Holarctic (Figures2b, and 3) (Almeida *et al.*, 2014). The Australasia population has diverged by about 4% from other populations, and isolates are reproductively isolated (up to 73% spore non-viability). In contrast, isolates found across a broad geographic range in the northern hemisphere are all remarkably closely related to each other within the South America A/Holarctic population. Many Northern hemisphere isolates also show signs of introgressions from *S. cerevisiae*, *S. kudriavzevii* and *S. eubayanus*, while isolates from the southern hemisphere do not (Almeida *et al.*, 2014). These patterns are evidence that *S. uvarum* from the South America A population recently colonized the northern hemisphere and were dispersed during the course of domestication.

## Conclusions and future directions

Domestication has dramatic consequences for evolution. Comparisons between domesticated and wild *Saccharomyces* show that population bottlenecks, high phenotypic diversity, low DNA sequence divergence, hybridization and introgression are all associated with domestication. Conclusions about genomic evolution drawn from *S. cerevisiae* must take its history of domestication into account. Close study of the entire *Saccharomyces* clade will allow us to identify general evolutionary mechanisms, as opposed to those that are the result of domestication.

Studying wild *Saccharomyces* will enable us to better understand the natural history of *S. cerevisiae* and how selective pressures have shaped its evolution. But the basic biology of *Saccharomyces* is still poorly understood. Are they active or dormant on bark or soil? How do they interact with other microbes? When, where and how often do they have sex? How do they disperse? These answers will not only improve the utility of *S. cerevisiae* as a model organism for fundamental biology, they will also allow the development of the genus *Saccharomyces* as a model ecological and evolutionary system.
